# Supplementary material for: Mutation analysis of multiple pilomatricomas in a patient with myotonic dystrophy type 1 suggests a DM1-associated hypermutation phenotype
Source: PLoS One. 2020 Mar 10;15(3):e0230003. doi: 10.1371/journal.pone.0230003 (PMC7064234; doi:10.1371/journal.pone.0230003)
Supplement: Attachment — Submitted filename: Response to Reviewers.pdf [file pone.0230003.s001.pdf]

UNIKLINIK
RWTHAACHEN

Klinik für Dermatologie
und Allergologie – Hautklinik

Uniklinik RWTH Aachen · Klinik für Dermatologie und Allergologie
Pauwelsstraße 30 · 52074 Aachen

Ruben Artero, Ph.D.
Academic Editor
PLOS ONE

Direktor
Univ.-Prof. Dr. med. Amir Yazdi
hautklinik-chefsekretariat@ukaachen.de

Universitätsklinikum Aachen
Anstalt öffentlichen Rechts (AöR)
Pauwelsstraße 30
52074 Aachen

www.hautklinik.ukaachen.de

Sekretariat
Silvia Kojtka
Tel.: 0241 80-88331
Fax: 0241 80-82413
skojtka@ukaachen.de
hautklinik-chefsekretariat@ukaachen.de

Leitender Oberarzt
Prof. Dr. med. Jens Malte Baron
jbaron@ukaachen.de

Oberärzte
Priv.-Doz. Dr. med. Verena von Felbert
vvonfelbert@ukaachen.de

Prof. Dr. med. Mosaad Megahed
mmegahed@ukaachen.de

Priv.-Doz. Dr. med. Mark Neis
mneis@ukaachen.de

Prof. Dr. med. Albert Rübben
albert.ruebben@post.rwth-aachen.de

Dr. med. Laurenz Schmitt
lschmitt@ukaachen.de

Funktionsoberärzte
Dr. med. Johanna Plange
jplange@ukaachen.de

Dr. med. Gerda Wurpts
gwurpts@ukaachen.de

Kliniksekretariat
Brigitte Lecoeur-von Cleef
Tel.: 0241 80-89564
Fax: 0241 80-82186
blecoeur@ukaachen.de

Privatambulanz
Tel.: 0241 80-80333
Fax: 0241 80-82185
terminvergabe-hautklinik@ukaachen.de

Poliklinik (Terminvergabe)
Tel.: 0241 80-80333
Fax: 0241 80-82185
terminvergabe-hautklinik@ukaachen.de

Tagesklinik
Tel.: 0241 80-88510
Fax: 0241 7501-480

Station DE01
Tel.: 0241 80-88356
Fax: 0241 7501-649

Termine zur stationären Aufnahme
Tel.: 0241 80-35655

Ihre Nachricht vom	Unser Zeichen	Durchwahl/Name	Datum
		36240/Prof. Dr. Rübben	1.2.2020

Dear Dr. Artero,

We would like to thank you for your interest in our manuscript and the helpful comments of the reviewers. A revised manuscript has been uploaded and we have tried to comply with all comments.

1) Have the authors made all data underlying the findings in their manuscript fully available? Reviewer #2: No

In order to share the raw NGS-data we have uploaded the data under: SRA: https://www.ncbi.nlm.nih.gov/sra SubmissionID: SUB6884814 BioProject ID: PRJNA603431 and included this information in the revised manuscript.

2) Reviewer #1: (i) The authors should tone down their conclusions, since samples are obtained from only individual, and focus more on presenting their results rather than confronting previous ideas and or hypothesis.

We agree to the reviewer and have soften author summary, introduction and discussion to this regard.

3) Reviewer #1: (ii) Authors should check whether Wnt pathway is activated in the different tumors checking expression of B-catenin and APC by immunohistochemistry and downstream targets such c-myc or cyclin D among others.

We have introduced immunohistochemistry for expression of CTNNB1, c-myc and cyclin D1 and could demonstrate that CTNNB1 is highly

Vorsitzender des Aufsichtsrates
Dr. Robert G. Gossink

Vorstandsvorsitzender
Prof. Dr. med. Thomas H. Ittel

Kaufmännischer Direktor
Dipl.-Kfm. Peter Asché

Sparkasse Aachen · BIC: AACSDE33
BLZ: 390 500 00 · Kto.: 13 004 015
IBAN: DE27 3905 0000 0013 0040 15
Commerzbank AG · BIC: DRESDEFF390
BLZ: 390 800 05 · Kto.: 203 309 400
IBAN: DE79 3908 0005 0203 3094 00
USt-IdNr.: DE813100566

expressed in the pilomatricoma which further supports a causative role of CTNNB1 mutations. This has already been described before in non-syndromic pilomatricoma. Expression of c-myc and cyclin D could be detected as well, but at a lower level. Nevertheless, lower expression of c-myc and cyclin D is consistent with pilomatricoma being a benign and very slowly growing tumor. Absence of expression of c-myc and cyclin D in shadow cells which do not express CTNNB1 further supports the assumption that WNT-CTNNB1-pathway plays a crucial role in pilomatricoma.

4) Reviewer #1: (iii) Results from NGS only found a mutation in ATM gene. I would have expected higher percentage. Do the authors have an explanation for this low number.

Indeed, this is a low number of detected mutations, however, in our experience this is not a totally unexpected finding for a panel that encompasses only about 0.4 Mb cumulative target size. The finding is also in line with published data on large tumor mutational burden studies. E.g. Chalmers et al. analyzed 100.000 human cancer genomes and showed that several cancer types show less than 1 mutation/Mb[1].

Moreover, our assumption is that there might be a transcriptional bias which favors mutation acquisition in CTNNB1 but does not lead to a high mutation load in the whole genome.

[1]Chalmers ZR, Connelly CF, Fabrizio D, Gay L, Ali SM, Ennis R, Schrock A, Campbell B, Shlien A, Chmielecki J, Huang F, He Y, Sun J, Tabori U, Kennedy M, Lieber DS, Roels S, White J, Otto GA, Ross JS, Garraway L, Miller VA, Stephens PJ, Frampton GM. Analysis of 100,000 human cancer genomes reveals the landscape of tumor mutational burden. Genome Med. 2017 Apr 19;9(1):34.

5) Reviewer #1: (iv) Authors should indicate whether pilomatricomas and non-syndromic pilomatricoma present mutations within the genes checked in NGS studies and discuss the differences with their study.

This would certainly be a very interesting scientific question that needs to be answered. However, to the best of our knowledge, no NGS data on pilomatricomas have been published to date.

6) Reviewer #2: The CTNNB1 mutations the authors have identified in the cancers of the myotonic dystrophy patients have been shown to sensitize tumor cells to TTK kinase inhibitors (Mol. Cancer Ther. 16(11)

2609-17). Do the authors believe that treatment with TTK inhibitors could be a viable option for these patients?

The correlation between overexpression of the assembly checkpoint kinase TTK (Mps1) and CTNNB1 mutations is not understood. It could be that Mps1 overexpression favors the occurrence of CTNNB1 mutations or that CTNNB1 overexpression by activating mutations enhances Mps1 expression. In the first case, we would not expect a role of TTK inhibitors in treating tumors of DM1 patients as we assume that CTNNB1 mutations are due to simultaneous transcription of CTNNB1 and the mutated DMPK gene. In the latter case, we could expect a role in treatment if the tumors of DM1 patients would acquire aneuploidy due to Mps1 overexpression, but this would only be the case in malignant tumors and not in pilomatricomas. We have not included this discussion in the revised manuscript.

7) Reviewer #2: Please provide more technical detail on how the relative fraction of tumor cells was determined in the samples that were sequenced in the Methods section. Please provide more details on the results of the sequence analysis, more specifically, the % of reads that were harboring the mutant vs. the wild-type CTNNB1 gene.

We have included the requested information in the manuscript.

8) Reviewer #3: I would like to know the clinical information of each tumor (size, location, etc.). Because multiple pilomatricoma in DM1 patients sometimes get larger than non-syndromic solitary pilomatricoma.

We have included the requested information in the manuscript.

9) Reviewer #3: Your hypothesis on interaction of toxic RNA from mutated DMPK gene is really interesting. I was just wondering if co-translation of DMPK gene and CTNNB1 gene has already been evidenced in the literature or not? Or is it just a hypothesis? I was not able to find any reference about that in this paper.

This is just our hypothesis. We replaced co-translation by simultaneous transcription in the manuscript as the putative interaction should already take place at the stage of transcription as described in figure 3 (2). We did not use the term co-transcription as this term is also used for specific forms of post transcriptional modifications. Nevertheless, co-transcription

UNIKLINIK
RWTHAACHEN
Klinik für Dermatologie
und Allergologie – Hautklinik

in the sense of simultaneous transcription has been proposed as a mechanism for gene fusions: Wright RL and Vaughan AT. A systematic description of MLL fusion gene formation.Crit Rev Oncol Hematol 2014 -Review. PMID 24787275. We have included this reference.

With best regards

Prof. Dr. med. Albert Rübben

Head of the Euregio Skin Cancer Center
